# RUNX2 and TAZ-dependent signaling pathways regulate soluble E-Cadherin levels and tumorsphere formation in breast cancer cells

**DOI:** 10.18632/oncotarget.4654

**Published:** 2015-07-20

**Authors:** Jessica L. Brusgard, Moran Choe, Saranya Chumsri, Keli Renoud, Alexander D. MacKerell, Marius Sudol, Antonino Passaniti

**Affiliations:** ^1^ Program in Molecular Medicine, University of Maryland School of Medicine, Baltimore, MD, USA; ^2^ Department of Medicine, University of Maryland School of Medicine, Baltimore, MD, USA; ^3^ Greenebaum Cancer Center, University of Maryland School of Medicine, Baltimore, MD, USA; ^4^ Department of Pharmaceutical Sciences, University of Maryland School of Pharmacy, Baltimore, MD, USA; ^5^ Mechanobiology Institute, Department of Physiology, National University of Singapore, Singapore; ^6^ Department of Pathology and Department of Biochemistry & Molecular Biology, The Marlene & Stewart Greenebaum Cancer Center, University of Maryland School of Medicine, Baltimore, MD, USA; ^7^ The Veteran's Health Administration Research & Development Service, Baltimore, MD, USA; ^8^ Department of Hematology/Oncology, Mayo Clinic, Jacksonville, FL, USA; ^9^ Laboratory of Genitourinary Cancer Pathogenesis, NCI, Bethesda, MD, USA

**Keywords:** breast cancer, transcription factors, tumorspheres, therapeutics

## Abstract

Intratumoral heterogeneity and treatment resistance drive breast cancer (BC) metastasis and recurrence. The RUNX2 transcription factor is upregulated in early stage luminal BC. However, the precise mechanism by which RUNX2 regulates an oncogenic phenotype in luminal BCs remains an enigma. We show that RUNX2 is predictive of poor overall survival in BC patients. RUNX2 associated with the TAZ transcriptional co-activator to promote a tumorigenic phenotype that was inhibited by knockdown of TAZ. RUNX2 increased endogenous TAZ translocation to the nucleus, which was prevented by inhibiting RUNX2. RUNX2/TAZ interaction was associated with ectodomain shedding of an oncogenic soluble E-Cadherin fragment (sE-Cad), which is known to cooperate with human epidermal growth factor receptor-2 (HER2/ErbB2) to increase BC growth. Neutralizing E-Cadherin antibodies or TAZ knockdown reduced the levels of sE-Cad in RUNX2-expressing BC cells and inhibited tumorsphere formation. RUNX2 expression also increased HER2-mediated tumorsphere size, which was reduced after treatment with the HER2-targeting agents Herceptin and lapatinib. These data support a novel role for RUNX2 in promoting an oncogenic phenotype in luminal BC in the context of TAZ, sE-Cad, and HER2. Using this signaling pathway to monitor BC cell oncogenic activity will accelerate the discovery of new therapeutic modalities to treat BC patients.

## INTRODUCTION

Breast cancer (BC) is a heterogeneous disease [1, 2] and despite advances in treatment, it remains the second leading cause of cancer-related deaths among women [3]. Luminal BC has the highest rates of relapse, often localizes to the bone [1, 4], and accounts for 50% of all metastatic-related BC deaths [5] in spite of the primary tumor being highly responsive to treatment. Given their high rate of relapse, it is clear that current treatment modalities are insufficient to completely eradicate these heterogeneous tumors. The HER2-targeting agent trastuzumab is only FDA-approved for use in patients whose tumors are clinically defined as HER2-enriched. However, early clinical trials have shown a 50% reduction in recurrence rates in patients with luminal BC treated with combination trastuzumab/chemotherapy over patients treated with chemotherapy alone [6]. Since ductal carcinomas *in situ* (DCIS) express HER2 prior to a transition to an invasive phenotype, there may be clinical benefit to treating BC with HER2-targeted agents even in the absence of *HER2* gene amplification.

RUNX2, an osteoblast differentiation transcription factor, is expressed in developing breast epithelial cells and is enriched in the mammary stem cell population responsible for terminal end bud differentiation [7, 8]. In basal-type breast cancer cell lines RUNX2 promotes an osteomimetic phenotype and metastasis to the bone through transcriptional activation of osteopontin, MMPs, and VEGF [9–11]. The RUNX2 oncogenic program can be stimulated through a variety of signaling pathways [12, 13] that include cooperation with TGFβ/Smad signaling [14–17]. RUNX2 is also expressed in early stage estrogen receptor positive (ER+) BC above normal levels found in the breast epithelia [18, 19]. However, its role in regulating luminal BC has yet to be elucidated. RUNX2 was recently shown to be upregulated in a subpopulation of luminal A MCF7 cells that share molecular characteristics with a more invasive BC phenotype, including genes associated with stem cell renewal and enhanced tumorsphere-forming capacity [20]. Whether it is a direct regulator of these tumorigenic programs was not determined. The RUNX2 binding partners, YAP (Yes-associated protein) [21] and TAZ (transcriptional co-activator with PDZ-binding motif) [22] are WW domain-containing transcriptional coactivators that promote cell transformation [23], osteogenesis [22], or stem cell self-renewal [24, 25]. TAZ is a nuclear effector of the Hippo tumor suppressor pathway that has been implicated in promoting BC progression [26], but its cooperative interaction with RUNX2 in BC has yet to be elucidated. Disruption of cell:cell contacts (Hippo pathway inactivation) results in reduced phosphorylation of TAZ leading to nuclear translocation and interaction with transcription factors that regulate expression of cell proliferation and anti-apoptotic genes [27]. TAZ is upregulated in 20% of BC patients [28] and is expressed in many breast cancer cell lines [26] where it has been shown to increase migration, invasion, tumorigenesis, drug resistance, and to promote an EMT [29]. TAZ and RUNX2 have been independently implicated in mediating metastasis to the bone [9, 30] but a cooperative role in BC has not been reported.

Although an epithelial-mesenchymal transition (EMT) in BC is characterized by downregulation of E-Cadherin [31–33], it is becoming increasingly clear that cells may also disseminate from the primary tumor without undergoing an EMT or down-regulating E-Cadherin expression [20, 34, 35]. An alternative pathway involving the proteolytic processing of the N-terminus of E-Cadherin (120 kDa), which results in the release of an ectodomain soluble oncogenic fragment (sE-Cad; 80 kDa), has been reported to mediate migration, invasion, and proliferation while maintaining epithelial morphology in cancer cells [35–40]. The proteolytic processing of E-Cadherin is regulated by matrix metalloproteinases (MMPs) and ‘A Disintegrin and Metalloproteinase s’ (ADAMs) including but not limited to MMP2, MMP9, and ADAM15 [35, 40–45]. MMPs and ADAM proteases secreted from tumor and stromal cells target full length E-Cadherin N-terminal of the transmembrane domain, resulting in the release of the intact extracellular domain. The remaining membrane-bound and intracellular domains have been shown to be further proteolytically processed, but, the function of these domains is poorly understood. sE-Cad is an autocrine and paracrine factor that promotes survival and metastatic progression by interacting with HER2/ErbB receptors [35, 38, 40, 46, 47]. In addition, sE-Cad binds full length E-Cadherin resulting in the destabilization of adherens junctions [35]. sE-Cad has been proposed as a functional metastatic biomarker in many cancers [35, 37, 39, 48, 49] including, but not limited to, BC [48]. We now report that RUNX2 expression in luminal BC cells results in nuclear TAZ localization and expression of sE-Cad. We found that TGFβ enhances the RUNX2-mediated expression of sE-Cad and upregulation of HER2. RUNX2 associated with TAZ in the nucleus and knockdown of TAZ inhibited RUNX2-mediated tumorsphere formation, which was also dependent on HER2 activation. Inhibition of RUNX2 with a novel RUNX2-targeting compound (CADD522) inhibited tumorsphere formation without affecting RUNX2 control cells. Furthermore, downregulation of RUNX2 levels with CADD522 inhibited sE-Cad production and TAZ nuclear localization in RUNX2 overexpressing cells. These results suggest that RUNX2:TAZ and activation of sE-Cad/HER2 signaling could be potential oncogenic pathways for a BC population of RUNX2 positive cells with metastatic potential.

## RESULTS

### RUNX2 expression and function in luminal BCs

**Figure 1:**
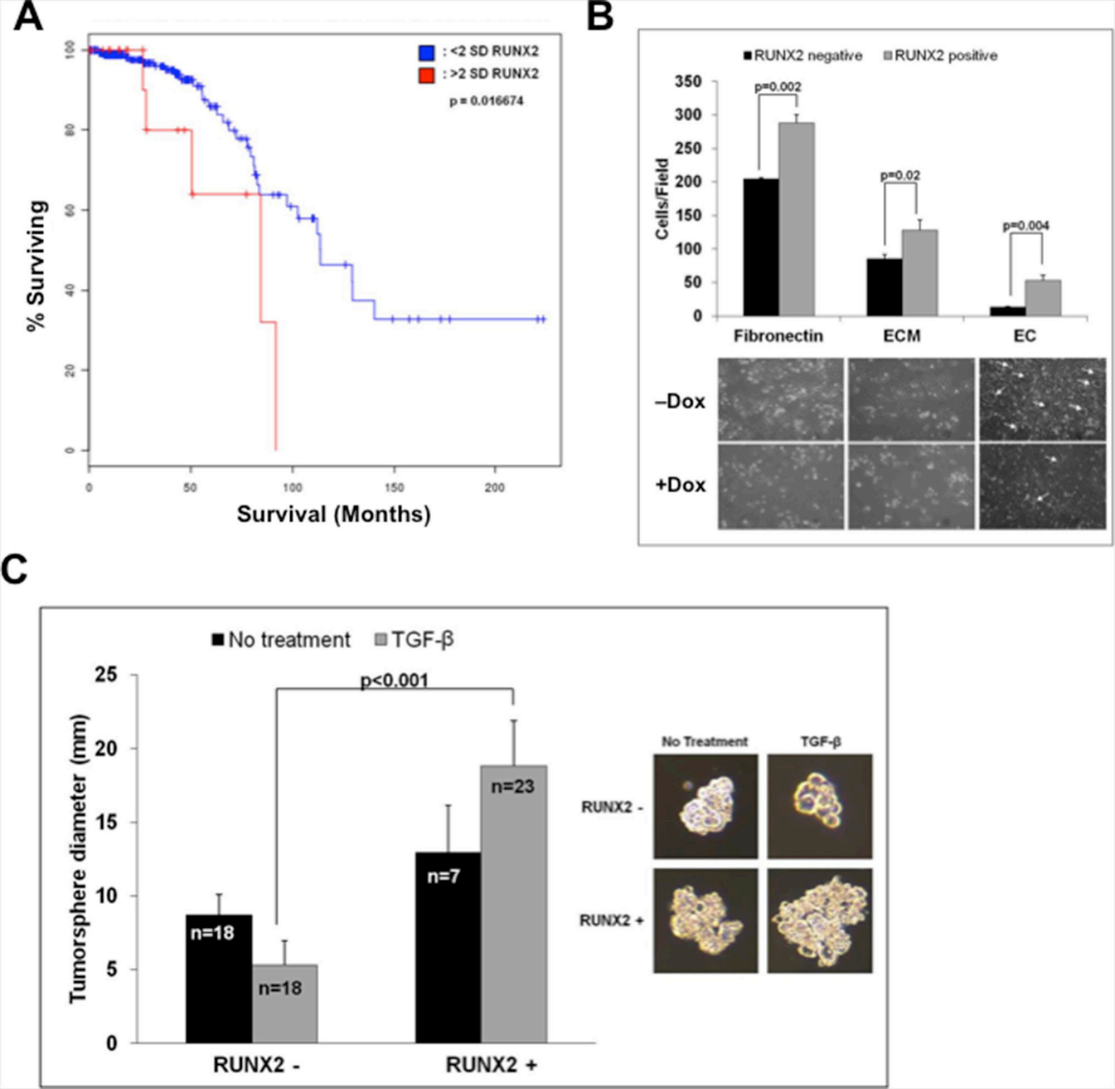
RUNX2 expression in luminal BCs and anchorage independent growth **A.** RUNX2 correlates with poor overall survival in patients with Luminal BC. Kaplan-Meier survival curves for RUNX2 expression in luminal BC patients were calculated from the Cancer Genome Atlas (TCGA). Results shown represent protein expression and Kaplan-Meier curves indicate % patients surviving (overall survival) as a function of months after diagnosis for patients with high RUNX2 protein expression (>2 SD) and patients with lower levels of RUNX2 protein (<2 SD). Low RUNX2 expression was significantly associated with median survival of 80 months compared to 117.5 months for high RUNX2 expression (*P* = 0.016). **B.** RUNX2 promotes attachment and invasion of RUNX2 overexpressing BC cells. Adhesion of MCF7 cells to tissue culture plates coated with fibronectin, extracellular matrix (ECM), or to a monolayer of endothelial cells (HBME cells) was measured 120 min after adding tumor cells. The number of attached cells/field was counted from 3–4 wells/field at high magnification (40X). Representative photographs depict MCF7 cell spreading 16 hr after adhesion. Arrows indicate areas where MCF7 tumor cells have invaded through the endothelial cell monolayer and attached to the underlying matrix. **C.** RUNX2 promotes tumorsphere formation. MCF7-RUNX2 Tet.OFF cells were scraped from culture dishes and plated in each well of a 6-well ultra-low attachment plate for 7 days. Cells were supplemented with (gray bars) or without (black bars) 2 ng/mL TGFβ. Tumorsphere diameter (mm) was calculated from photographic images using the formula (L+W)/2. The number of colonies measured is indicated in each bar graph and designated by “*n*”. Statistical analysis (Student's *t*-test or ANOVA) was used to determine significance between RUNX2 overexpressing or RUNX2 control treatment groups. Representative photos of colonies are shown.

To test the hypothesis that RUNX2 might regulate the tumorigenic phenotype of luminal BC cells, RUNX2 expression was examined in patients diagnosed with early stage luminal BC (Figure 1A). RUNX2 protein expression was associated with poor prognosis after diagnosis (overall survival; *P* = 0.017) in those patients with high RUNX2 levels (>2 SD; median survival 80 months) compared to patients with lower levels of RUNX2 protein (<2 SD; median survival 117.5 months). To define models of luminal BC, several cell lines were examined for RUNX2 expression. Variable levels of endogenous RUNX2 protein were detectable in luminal MCF7, HCC1428, and T47D BC cells (Supplementary Figure S1A). To examine its oncogenic function, we expressed RUNX2 in the luminal BC cell line, MCF7, under the control of a Tet.OFF promoter (Supplementary Figure S1A). RUNX2 expression in the MCF7 Tet.OFF model increased attachment to fibronectin, extracellular matrix, and increased invasion through an endothelial cell (EC) monolayer (Figure 1B). Culture of BC cells in suspension is a model of anchorage-independent growth and may define a transformed BC cell phenotype (tumorspheres) [50]. We showed recently that shRNA knockdown of RUNX2 in triple negative BC cells inhibited tumorsphere formation [51]. Consistent with these results, inducible RUNX2 expression (doxycycline –) in MCF7 cells resulted in tumorspheres that were significantly larger (3.6-fold; *p* < 0.001) than MCF7 cells in which RUNX2 expression was repressed (doxycycline +) especially in the presence of TGFβ (Figure 1C). It is well-established that TGFβ exhibits both tumor suppressive and tumor promoting activities. In the presence of an oncogenic factor, TGFβ functions to promote tumor growth; while in most normal cells (absence of an oncogenic factor) TGFβ inhibits cell proliferation and restrains tumor formation [26]. Smad proteins, downstream nuclear effectors of TGFβ signaling, are also known to cooperate with RUNX2 to potentiate tumorigenesis. Treatment of RUNX2 overexpressing MCF7 cells with TGFβ resulted in larger tumorspheres (18.83 ± 3.08) relative to control cells expressing low RUNX2 (5.31 ± 3.21) (Figure 1C). In fact, in the absence of RUNX2 overexpression, MCF7 control cells were inhibited by TGFβ treatment, as evident by a decrease in tumorsphere diameter (8.69 ± 1.41 relative to 5.31 ± 1.68) (Figure 1C). Therefore, in luminal BC cells, RUNX2 mediates an oncogenic phenotype that is characterized by increased attachment to extracellular matrix, invasion, and anchorage-independent growth.

**Figure 2:**
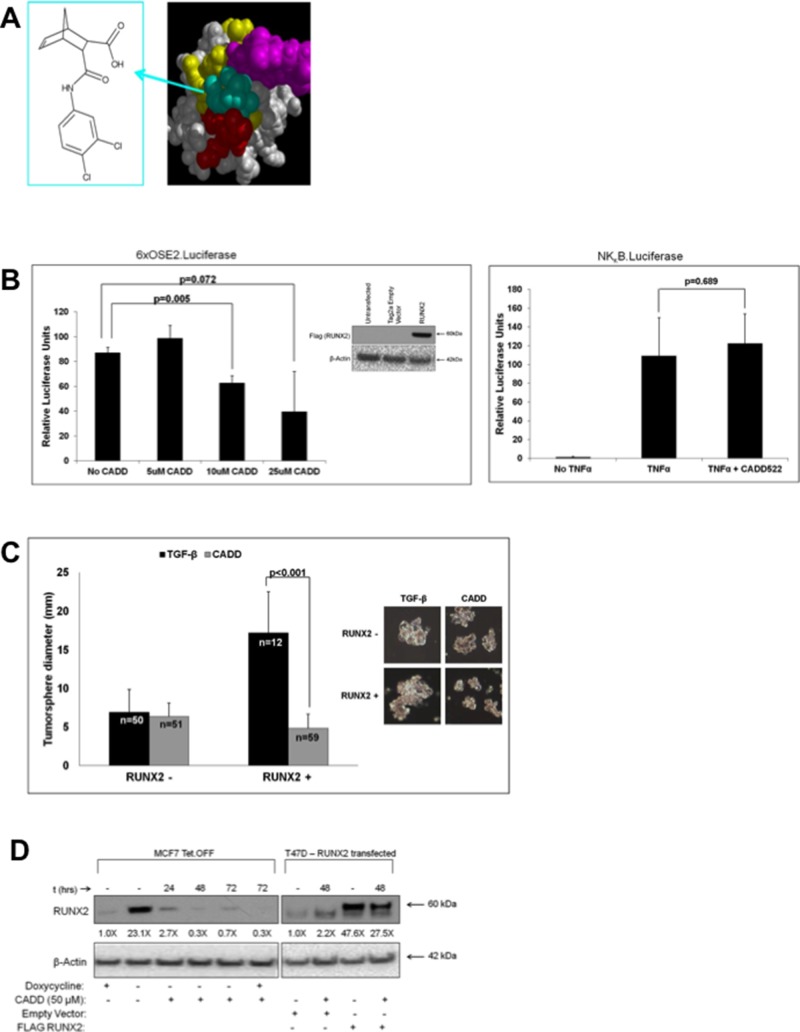
RUNX2-targeting compound CADD522 inhibits RUNX2-positive MCF7 tumorsphere formation in suspension **A.** The CADD522 compound *(left panel*) was identified from a computer-assisted drug design screen and validated in DNA binding assays to inhibit RUNX2 binding to its cognate DNA-binding domain [52]. It exhibits an IC50 = 10 nM in D-ELISA DNA binding assays [52]. A best-fit model (*right panel*) predicts interaction with the tail (purple spheres), wing (red spheres), and other base residues (yellow) of the RUNX2 DNA-binding (Runt) domain. **B.** CADD522 inhibits RUNX2 transcriptional activity. NIH3T3 cells were seeded into 24-well culture dishes at 30,000 cells/well. Cells were allowed to recover for 24 hours and then transfected with 100 ng RUNX2 or Tag2a empty vector plus 100 ng 6xOSE2.Luciferase and 10 ng pTK Renilla (left panel). At the time of transfection cells were treated with different doses of CADD522 and incubated for 24 hours. Relative Luciferase Units were calculated after normalization to Renilla activity and represent the difference between RUNX2 and Tag2a empty vector transfection (*inset Western blot, left panel*). NIH3T3 cells were also cultured in 24-well culture dishes at 30,000 cells/well and allowed to recover for 24 hours before transfection with 100 ng NFκB.Luciferase and 10 ng pTK Renilla (right panel). Cells were treated overnight with the NFκB signaling activator, TNFα (20 ng/mL) or TNFα (20 ng/mL) and 10 μM CADD522. Relative Luciferase Units are shown and *t*-test were performed to determine statistical significance (as indicated). **C.** CADD522 inhibits RUNX2 overexpressing MCF7 tumorsphere formation. MCF7-RUNX2 Tet.OFF cells were plated in each well of a 6-well ultra-low attachment plate with 2 ng/mL TGFβ for 12 days. Cells were treated with (gray bar) or without (black bar) 50 μM CADD522. Tumorsphere diameter (mm) was calculated from photographic images. The number of colonies measured is indicated in each bar graph and designated by “*n*”. Representative photos of colonies are shown. **D.** CADD522 inhibits RUNX2 expression. MCF7-RUNX2 Tet.OFF cells were grown in the presence (RUNX2-) or absence (RUNX2+) of doxycycline and treated with 50 μM CADD522. In parallel, a low RUNX2-expressing luminal BC cell, T47D (Supplementary Figure S1A), was transfected with either an empty vector (ctrl) or a FLAG-tagged RUNX2-expressing vector and treated with 50 μM CADD522 for 48 hr. Nuclear proteins were collected and resolved by SDS-PAGE. RUNX2 protein levels were quantified using NIH ImageJ and normalized to β-actin. Fold-changes relative to untreated cultures are indicated below each lane.

To validate that tumorsphere growth was RUNX2 dependent, cells were treated with an inhibitor of RUNX2:DNA binding (Figure 2A) that was discovered by computer assisted drug design (CADD) to interact with the RUNX2 DNA-binding pocket [52]. The CADD522 compound was able to dose dependently inhibit transcriptional activity of a RUNX2 regulated promoter, 6XOSE2. Luciferase, while having no effect on an unrelated NFκB.Luciferase reporter gene activated by TNFα (Figure 2B). RUNX2 overexpressing MCF7 cells were treated with CADD522 in suspension. This RUNX2 inhibitor significantly decreased the diameter of RUNX2 overexpressing MCF7 tumorspheres (17.21 ± 5.28 to 4.83 ± 1.87; *p* < 0.001) by almost 4-fold relative to vehicle-treated cells (Figure 2C). RUNX2 control cells were unaffected (6.98 ± 2.89 to 6.37 ± 1.78; *P* = 0.209). Therefore, RUNX2 is necessary for luminal BC cell tumorsphere formation. RUNX2 protein stability is regulated by phosphorylation, which promotes DNA binding [53, 54]. When RUNX2 is not bound to DNA, it is either targeted to sub-nuclear compartments [55] or rapidly degraded [56]. To determine whether inhibiting RUNX2 DNA binding and transcriptional activity altered the levels of RUNX2 we treated RUNX2 overexpressing MCF7 cells or T47D cells overexpressing RUNX2 with the CADD522 compound. CADD522 reduced RUNX2 protein levels in both MCF7 and T47D cells (Figure 2D). In MCF7 cells, RUNX2 protein levels were reduced about 8-fold after 24 hr treatment (reduction of 90%) and greater than 95% after 48 hr. In RUNX2 overexpressing MCF7 cells (doxycycline –) that were re-treated with doxycycline (doxycycline +) to repress RUNX2 expression (Supplementary Figure S1B, *upper panel*), CADD522 was able to further reduce RUNX2 protein levels below control cells (Figure 2D, *lane 6*). Untreated RUNX2 overexpressing cells (doxycycline –) maintained high levels of RUNX2 over a 72 hr period (Supplementary Figure S1B, *lower panel*). In T47D cells, RUNX2 protein levels were reduced about 2-fold after 48 hr confirming that targeting RUNX2 DNA binding can alter RUNX2 levels *in vivo*. In summary, the ability of luminal BC cells to form tumorspheres is dependent on RUNX2 since tumorsphere size was reduced in cells treated with a compound that inhibits RUNX2 protein levels, DNA binding, and transcriptional activity.

### Role of RUNX2 cofactor TAZ in anchorage independent growth

**Figure 3:**
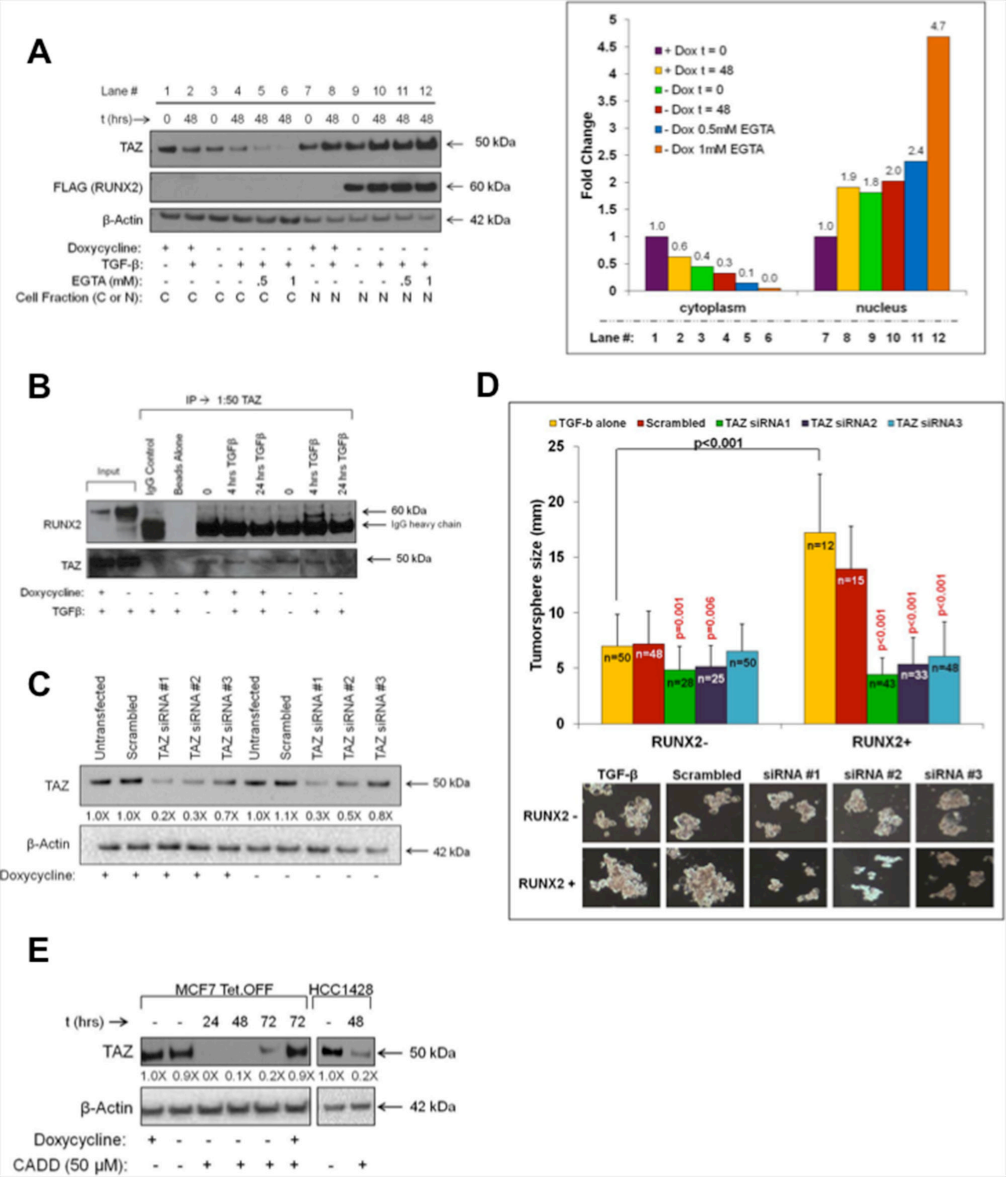
TAZ cooperates with RUNX2 to promote tumorsphere formation **A.** RUNX2 promotes TAZ nuclear translocation. MCF7-RUNX2 Tet.OFF cells were grown in the presence (RUNX2–) or absence (RUNX2+) of doxycycline for 3 days and starved 16 hr in D5030 supplemented with 1 mM glucose and 2% FBS (t = 0). Cells were then treated for 48 hr (t = 48) with 2 ng/mL TGFβ with or without EGTA (0.5 mM or 1 mM). Cytoplasmic and nuclear extracts were resolved by SDS-PAGE and immunoblots were probed with antibodies for YAP/TAZ (50 kDa), FLAG (RUNX2, 60 kDa), and β-Actin (42 kDa). Cytoplasmic and nuclear TAZ (50 kDa) protein bands were normalized to β-Actin and quantified using NIH Image-J. Fold-changes are shown. **B.** RUNX2 and TAZ associated in the same immune complex. MCF7-RUNX2 Tet.OFF cells were grown in the presence (RUNX2–) or absence (RUNX2+) of doxycycline for 3 days and then nutrient deprived in D5030 media supplemented with 1 mM glucose and 2% FBS for 16 hr (t = 0) followed by treatment with 2 ng/mL TGFβ for 4 and 24 hrs. Nuclear lysates (400 μg) were immunoprecipitated (IP) using YAP/TAZ antibody, resolved by SDS-PAGE, and immunoblots were probed for RUNX2 and TAZ. To visualize TAZ expression a conformation-specific Rabbit IgG was used. Rabbit IgG and beads alone were used as controls. Inputs are nuclear lysates. **C.** TAZ knockdown in MCF7-RUNX2 Tet.OFF cells. MCF7-RUNX2 Tet.OFF cells were treated with siRNA targeting three different regions of the TAZ mRNA or scrambled siRNA control. Protein levels of TAZ (50 kDa) were assayed 96 hr post-transfection from whole cell lysates (RIPA). **D.** Knockdown of TAZ protein inhibits tumorsphere formation. MCF7-RUNX2 Tet.OFF cells were transfected with TAZ siRNA or scrambled control and 24 hr post transfection cells were scraped from culture dishes and plated into ultra-low attachment plates with 2 ng/mL TGFβ for 12 days. Wells were photographed and tumorsphere sizes were measured and calculated from photographic images (TGFβ alone = yellow bars; scrambled siRNA = red bars; siRNA#1 = green bars; siRNA#2 = purple bars; siRNA#3 = blue bars) using the formula: (L+W)/2. The number of colonies measured is indicated in each bar graph and designated by “*n*”. Representative photos of colonies are shown. **E.** CADD522 inhibits TAZ nuclear localization. MCF7-RUNX2 Tet.OFF cells were grown in the presence (RUNX2–) or absence (RUNX2+) of doxycycline. HCC1428 luminal BC cells were grown in RPMI. Cells were treated with 50 μM CADD522 for 24, 48, or 72 hr. Nuclear proteins were collected using the High/Low salt extraction method and resolved by SDS-PAGE. Proteins were visualized using antibodies against YAP/TAZ (50 kDa) and β-actin (42 kDa). TAZ protein bands were quantified using NIH Image-J and normalized to β-actin. Fold changes relative to untreated cultures are indicated. TAZ nuclear protein levels were unchanged in CADD522-treated RUNX2 control cells (MCF7 + doxycycline; 72 hr).

The Hippo signaling effectors, TAZ and YAP, are important RUNX2 transcriptional cofactors that interact with the coactivator/corepressor domains located in the C-terminus of RUNX2 [23]. YAP expression in RUNX2 overexpressing MCF7 cells was low and levels in response to RUNX2 did not change in these cells (Supplementary Figure S1C). However, nuclear TAZ levels were 80% higher upon induction of RUNX2 expression (doxycycline –) compared to control cells (doxycycline +) (Figure 3A, compare lane 9 to 7). Disruption of cell:cell contacts with EGTA resulted in additional increases in TAZ nuclear levels (lanes 11 and 12) with a concomitant decrease in cytosolic TAZ (lanes 5 and 6). Immunoprecipitation of TAZ showed that RUNX2 and TAZ were associated within the same immune complex only in RUNX2 overexpressing MCF7 cells treated with TGFβ (Figure 3B). To determine if RUNX2 and TAZ cooperatively promote tumorsphere formation, RUNX2 Tet.OFF MCF7 cells were transfected with scrambled siRNA or three specific siRNAs targeting TAZ. TAZ knockdown to levels 70–80% (siRNA1), 50–70% (siRNA2) or 20–30% (siRNA3) of control (scrambled siRNA) was observed in cells expressing either low (doxycycline +) or high (doxycycline –) RUNX2 (Figure 3C). Tumorsphere formation of RUNX2 overexpressing MCF7 cells (doxycycline –) was inhibited by all three TAZ-specific siRNAs over 12 days (Figure 3D). Although significant (*p* = 0.001 and *p* = 0.006), TAZ siRNA#1 (4.85 ± 2.11) and siRNA#2 (5.16 ± 1.91) knockdown produced a modest decrease in tumorsphere size compared to controls (6.97 ± 2.89 for TGFβ treated and 7.18 ± 2.98 for scrambled) in RUNX2 control cells (doxycycline +). TAZ siRNA#3 had no significant effect in the control cells. However, TAZ siRNA #1, 2, and 3 (4.43 ± 1.47, 5.35 ± 2.42, and 6.05 ± 3.12 respectively) inhibited tumorsphere formation 2.5 to 4-fold in the RUNX2 overexpressing MCF7 cells compared to controls (17.21 ± 5.28 and 13.97 ± 3.81 for TGFβ and scrambled controls respectively). Interestingly, a 20% reduction in TAZ protein levels (Figure 3C) was sufficient to significantly decrease tumorsphere formation by 65% in RUNX2 overexpressing cells (*p* < 0.001) while having no effect in RUNX2 control cells. Further, reducing RUNX2 expression in the RUNX2 overexpressing MCF7 (doxycycline –) cells with the CADD522 compound (Figure 2D) lowered TAZ nuclear levels within 24 hr (Figure 3E; doxycycline –). However, CADD522 treatment of RUNX2 overexpressing MCF7 cells (doxycycline –) that were re-treated with doxycycline (doxycycline +) to repress RUNX2 expression (Supplementary Figure S1B, *upper panel*) had no effect on nuclear TAZ levels over a 72 hr time course (Figure 3E, *lane 6*). This suggests that the decline in TAZ levels upon CADD522 treatment requires RUNX2 expression. Similarly, in HCC1428 BC cells expressing endogenous RUNX2, treatment with CADD522 led to an 80% reduction in nuclear TAZ levels compared to vehicle-treated controls (Figure 3E, *HCC1428*). In summary, RUNX2 interaction with TAZ is necessary to promote TAZ nuclear translocation and/or retention and tumorsphere formation.

### Production of oncogenic E-Cadherin ectodomain (sE-Cad) is dependent on RUNX2 and TAZ

**Figure 4:**
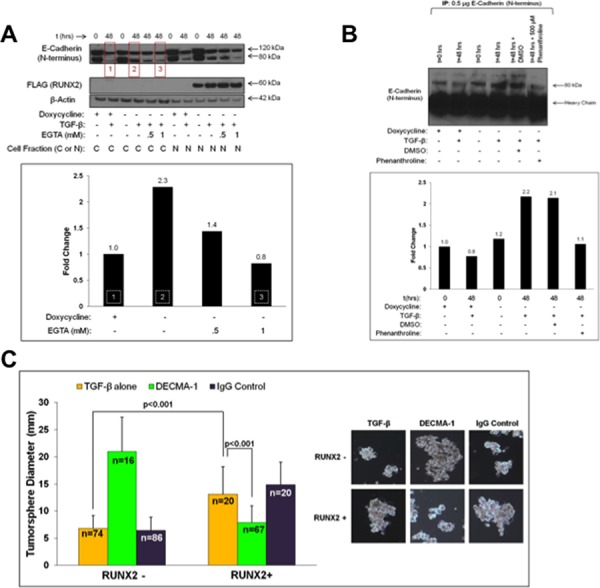

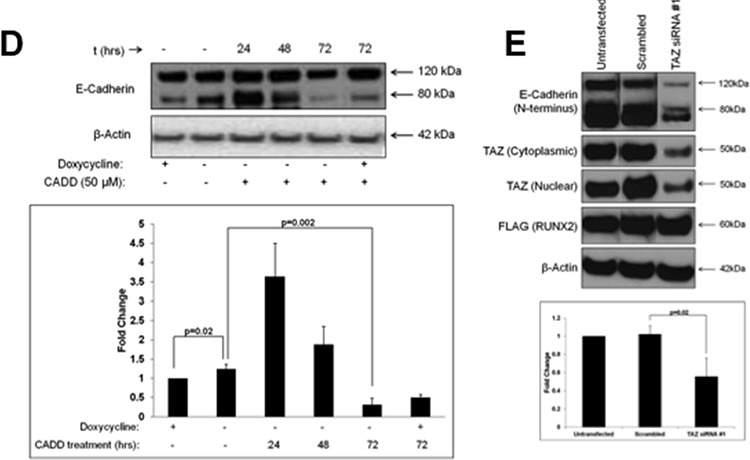
RUNX2 expression is associated with production of soluble E-Cadherin (sE-Cad) and tumorsphere formation **A.** RUNX2 increases the production of sE-Cad associated with the cell surface in response to TGFβ. MCF7-RUNX2 Tet.OFF cells were grown in the presence (RUNX2–) or absence (RUNX2+) of doxycycline for 3 days. Cells were nutrient deprived for 16 hr in D5030 supplemented with with 1 mM glucose and 2% FBS (t = 0) and then treated for 48 hr with 2 ng/mL TGFβ with or without EGTA (0.5 mM or 1 mM). Cytoplasmic and nuclear fractions were analyzed by western blot and probed with antibodies for E-Cadherin (120 kDa = full length; 80 kDa = sE-Cad), FLAG (RUNX2; 60 kDa), and β-Actin (42 kDa). Changes in sE-Cad protein levels were determined by quantifying the lower E-Cadherin band using NIH ImageJ normalized to β-actin. Red boxes in the gel correspond to the same numbered white-hashed boxes in the graph. Fold change values are indicated above corresponding bars. **B.** RUNX2 overexpressing MCF7 cells secrete higher levels of sE-Cad. Conditioned media from MCF7-RUNX2 Tet.OFF cells were collected following a 16 hr starvation in D5030 supplemented with 1 mM glucose and 2% FBS (t = 0) or after treatment with 2 ng/mL TGFβ for 48 hr with or without 500 μM o-Phenanthroline. Conditioned media were immunoprecipitated using E-Cadherin antibody. Proteins were eluted from beads and separated by SDS-PAGE followed by Western blot with antibodies to detect full-length (120 kDa) and sE-Cad (80 kDa). Changes in sE-Cad protein levels were determined by quantifying the lower E-Cadherin band using NIH ImageJ. Protein levels are expressed as fold change relative to RUNX2 control MCF7 cells (+ dox; – TGFβ). **C.** sE-Cad mediates tumorsphere formation in RUNX2 overexpressing MCF7 cells. MCF7-RUNX2 Tet.OFF cells were plated in 6-well ultra-low attachment plates with 2 ng/mL TGFβ. Cells were then treated with DECMA-1 (green bars) or IgG control (purple bars) or untreated (yellow bars) for 10 days. Cells were photographed and tumorsphere sizes were measured from photographic images (mm) and diameters graphed. Representative photos of colonies are shown to the right of the graph. Tumorsphere sample size is indicated in each bar as “*n*”. **D.** CADD522 treatment inhibits sE-Cad production. MCF7-RUNX2 Tet.OFF cells were grown in the presence (RUNX2-) or absence (RUNX2+) of doxycycline for 3 days. Cells were then treated with 50 μM CADD522 over a 72 hour time period. Cytoplasmic fractions were obtained and analyzed by western. Proteins were visualized using antibodies against E-Cadherin (Full length = 120 kDa; sE-Cad = 80 kDa) and β-actin (42 kDa). Western blots were run in triplicate and results were quantified using NIH ImageJ and normalized to β-actin. **E.** TAZ knockdown inhibits the production of sE-Cad. RUNX2 overexpressing MCF7 cells were grown in absence of doxycycline and than transfected with TAZ siRNA#1 (Figure 3C) to reduce TAZ levels. Cells were re-plated 24 hours post-transfection and allowed to re-attach for 72 hours. Cytoplasmic and nuclear extracts were obtained and the expression of E-Cadherin (120 kDa and 80 kDa), TAZ (cytoplasmic or nuclear) and RUNX2 (FLAG) was determined by Western blot. Changes in sE-Cad protein levels from 3 separate gels were determined using NIH ImageJ and normalizing to β-actin.

The EMT can be regulated by RUNX2 in some BC cells [57]. However, in luminal BC cells, RUNX2 did not promote the loss of E-Cadherin, the downregulation of ER or the expression of N-Cadherin and Vimentin, which are indicative of an EMT progression (Supplementary Figure S1D). Instead, RUNX2 expression was associated with a 129% increase in oncogenic E-Cadherin fragment, sE-Cadherin (sE-Cad; 80 kDa) consisting of the E-Cadherin ectodomain (Figure 4A). RUNX2 overexpressing MCF7 cells expressed 2.3-fold more sE-Cad in response to TGFβ treatment (48 hr), compared to RUNX2 control MCF7 cells (Figure 4A, *boxes 1 and 2*). sE-Cad protein levels were reduced after treatment with the Ca^+2^-chelator EGTA suggesting that sE-Cad was associated with the cell surface. sE-Cad is an established biomarker for metastatic prostate cancer [37, 39] and has been found in the conditioned media of DU145 prostate cancer cells where it promotes tumorigenesis and mediates invasion [37]. Conditioned media from MCF7 cells expressing RUNX2 contained 2.2-fold higher levels of sE-Cad ectodomain compared to RUNX2 control cells (Figure 4B). In contrast to cells overexpressing RUNX2, after 48 hr treatment with TGFβ there was a 20% reduction in sE-Cad secreted into the conditioned media by RUNX2 control cells. Baseline levels of sE-Cad released by the MCF7 Tet.OFF cells were similar in RUNX2 overexpressing and RUNX2 control cells at *t* = 0. Generation of sE-Cad is dependent upon the expression of MMPs and ADAM proteases [35]. RUNX2 has been shown to control transcription of numerous MMP genes and extracellular matrix molecules [10]. Relative to control MCF7 cells, RUNX2 overexpressing cells showed several-fold enrichment for MMP genes (Supplementary Figure S1E), some of which (MMP2) are known to target E-Cadherin for proteolytic processing. In addition, the expression of other MMPs and ADAM proteases was also upregulated in RUNX2 overexpressing cells (MMP11, MMP12, MMP16, and ADAMTS8). Interestingly, the expression of extracellular matrix genes laminin and collagen I (a RUNX2 target) were increased while expression of the anti-tumor and anti-angiogenic matrix protein thrombospondin was repressed. To determine if the production and secretion into the conditioned media of sE-Cad in RUNX2 overexpressing MCF7 cells was dependent upon the activity of MMPs, we treated cells with the broad spectrum MMP inhibitor, o-Phenanthroline. In the presence of o-Phenanthroline, sE-Cad levels were decreased to the levels present in RUNX2 control cells (Figure 4B). E-Cadherin and sE-Cad are neutralized using the ectodomain-specific E-Cadherin antibody, DECMA-1 [46]. To determine if sE-Cad mediated RUNX2-dependent tumorsphere formation, we treated RUNX2 overexpressing MCF7 cells with DECMA-1. Tumorsphere size was significantly inhibited in RUNX2 overexpressing (doxycycline –) cells treated with DECMA-1 over 10 days (7.85 ± 3.11; *p* < 0.001) compared to TGFβ and IgG controls (13.06 ± 5.16 and 14.88 ± 4.14 respectively) indicating RUNX2 overexpressing cells rely on sE-Cad for tumorsphere formation (Figure 4C). However, DECMA-1 promoted larger tumorsphere formation in RUNX2 control (doxycycline +) cells, perhaps through its ability to prevent full length E-Cadherin-dependent growth suppression, thus increasing cell proliferation, which is consistent with published reports [58]. To determine whether inhibition of RUNX2 protein levels and transcriptional activity would prevent the generation of sE-Cad, MCF7 Tet.OFF cells were treated with the RUNX2-selective drug CADD522. CADD522 treatment increased sE-Cad production 3.5-fold within the first 24 hr of treatment, but sE-Cad levels declined almost 20-fold by 72 hr in RUNX2 overexpressing cells (Figure 4D). Baseline levels of sE-Cad were significantly higher in RUNX2 overexpressing (doxycycline –) cells prior to CADD522 treatment (*P* = 0.02). A 2-fold decrease in sE-Cad production in RUNX2 control cells (doxycycline +) (Figure 4D) may be due to CADD522 inhibition of endogenous RUNX2 or of low amounts of RUNX2 produced by the Tet.OFF system even in the presence of doxycycline (Figure 2D; Supplementary Figure S1A). Further, siRNA-targeted knockdown of the RUNX2 cofactor TAZ also resulted in a 50% reduction (*p* = 0.02) of sE-Cad ectodomain levels without affecting RUNX2 levels (Figure 4E). Therefore, MCF7 cells produce endogenous sE-Cad, its levels can be modulated by the expression of RUNX2 and TAZ, and expression of sE-Cad ectodomain mediates tumorsphere formation in RUNX2-expressing luminal BC cells.

### Therapeutic targeting of sE-Cad/HER2 signaling in RUNX2-expressing luminal BC cells

**Figure 5:**
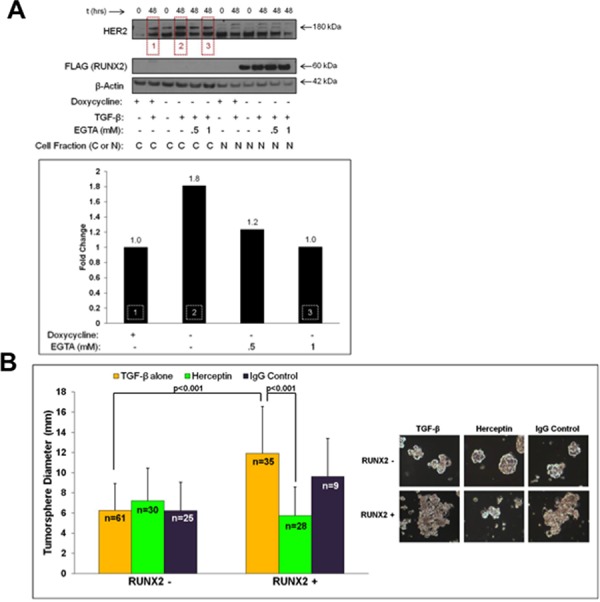

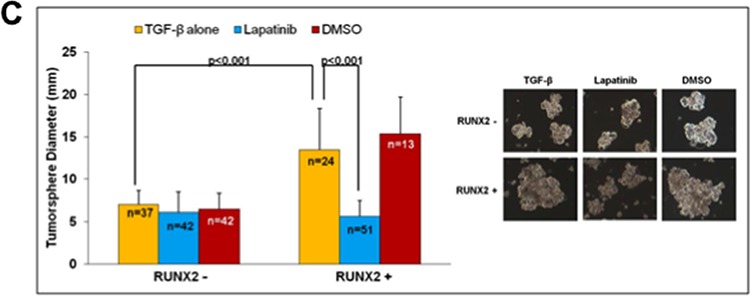
HER2 expression in RUNX2 positive cells that express elevated sE-Cad levels sensitizes cells to HER2-targeted drugs **A.** RUNX2 overexpressing MCF7 cells express higher HER2 protein levels in the response to TGFβ. MCF7-RUNX2 Tet.OFF cells were grown in the presence (RUNX2–) or absence (RUNX2+) of doxycycline for 3 days and starved for 16 hr in D5030 supplemented with 1 mM glucose and 2% FBS (t = 0). Cells were then treated for 48 hr with 2 ng/mL TGFβ with or without EGTA (0.5 mM or 1 mM). Cytoplasmic and nuclear fractions were obtained and proteins analyzed by Western blot. Antibodies for HER2 (180 kDa), FLAG (RUNX2; 60 kDa), or β-Actin (42 kDa) were used to visualize proteins. Changes in HER2 protein levels were determined using NIH ImageJ and normalizing to β-actin. Red boxes within the gel correspond to the same numbered white-hashed boxes on the graph. Fold change values are indicated above corresponding bars. **B.** RUNX2 overexpressing cells are sensitive to Herceptin. MCF7-RUNX2 Tet.OFF cells were plated in each well of a 6-well ultra-low attachment plate with 2 ng/mL TGFβ. Cells were left untreated (yellow bars) or treated with Herceptin (green bars) or IgG control (purple bars) for 10 days. Wells were replenished with Herceptin or IgG control every 2–3 days. Each well was photographed and tumorsphere diameters were measured from these images. Representative photos of colonies are shown. Tumorsphere sample size is indicated in each bar as “*n*”. **C.** RUNX2 overexpressing cells are sensitive to Lapatinib treatment. MCF7-RUNX2 Tet.OFF cells were plated in each well of a 6-well ultra-low attachment plate with 2 ng/mL TGFβ. Cells were then left untreated (yellow bars), or treated with lapatinib (blue bars), or DMSO control (red bars) for 15 days. After growth for 15 days, wells were photographed and tumorsphere diameters were measured from photographic images. Representative colonies are shown. Tumorsphere sample size is indicated in each bar as “*n*”.

The ErbB2/HER2 receptor family member is expressed in a subset of luminal BC cells in the absence of gene amplification [6] and HER2 is one of the main targets of sE-Cad [59]. HER2 interacts with sE-Cad to promote ligand-independent cell signaling in HER2-enriched BC cells, but its role in luminal BCs (in the absence of gene amplification) is undefined [1, 2, 60]. We showed recently that overexpression of RUNX2 increased Akt phosphorylation in MCF7 cells [51]. Since HER2 is known to activate downstream signaling involving Akt [59, 61], the levels and tumorsphere-forming potential of HER2 in RUNX2 overexpressing MCF7 cells was determined. After 48 hr treatment with TGFβ, RUNX2 overexpressing MCF7 cells contained almost 2-fold higher HER2 levels than RUNX2 control cells (Figure 5A, *boxes 1 and 2*) suggesting that RUNX2-mediated production of secreted sE-Cad (Figure 4A) may stabilize HER2. Inhibition of sE-Cad levels at the cell surface with EGTA (E-Cadherin destabilization to remove sE-Cad bound at the cell surface; Figure 4A, *box 3*) correlated with a decrease in HER2 to levels observed in RUNX2 control cells (Figure 5A, *box 3*). To test for functional HER2, the effect of HER2-targeted agents on tumorsphere formation was determined. The Herceptin monoclonal antibody binds the extracellular domain of HER2, promotes receptor internalization and degradation, prevents homo/heterodimerization, and mediates antibody-dependent cellular cytotoxicity [60]. Herceptin inhibited tumorsphere formation in RUNX2 overexpressing MCF7 cells by 2-fold (11.91 ± 4.65 to 5.76 ± 2.83; *p* < 0.001), without affecting tumorsphere formation in RUNX2 control cells (6.25 ± 2.68 to 7.2 ± 3.27; *P* = 0.145) (Figure 5B). Isotype-matched IgG had no impact on tumorsphere size. Lapatinib is a small molecule receptor tyrosine kinase inhibitor that interacts with the intracellular kinase domain of HER2 to inhibit receptor activation [60]. RUNX2 overexpressing MCF7 cells were sensitive to lapatinib treatment, with tumorsphere size reduced by 2.5-fold relative to vehicle control over 15 days (13.48 ± 4.87 to 5.68 ± 1.78; *p* < 0.001) (Figure 5C). Lapatinib did not affect tumorsphere formation of RUNX2 control cells (6.99 ± 1.67 to 6.08 ± 2.38). These results are consistent with a cooperative function for HER2 and sE-Cad in promoting a tumorigenic phenotype in luminal BC cells expressing RUNX2.

## DISCUSSION

**Figure 6:**
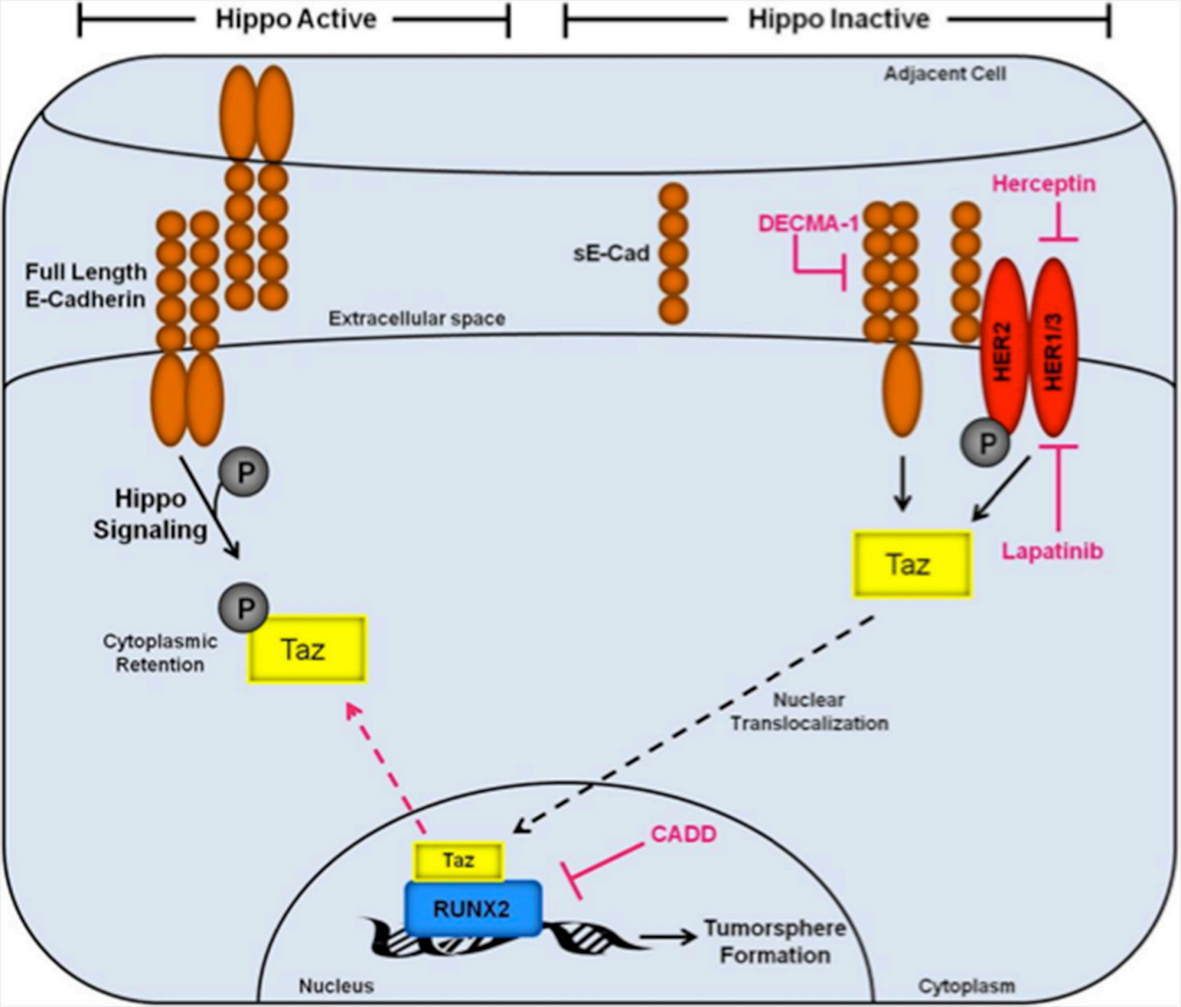
Inactivation of the Hippo tumor suppressor pathway by RUNX2 promotes a tumorigenic phenotype in luminal BC cells The Hippo tumor suppressor pathway is active (left side) in the context of s-Cadherin interactions that promote epithelial cell:cell polarity. Under these conditions, E-Cadherin signaling maintains TAZ phosphorylation (gray circle) and localization in the cytosol. Upon induction of oncogenic events (right side), which include sE-Cad production and cooperation with HER2 receptors, the Hippo pathway is inactivated promoting TAZ translocation (black hashed arrow) to the nucleus where it can interact with transcription factors, such as RUNX2, which are responsible for activation of genes that promote cell invasion, survival, and tumorsphere formation. Several therapeutic targets are indicated (pink) that inhibit the tumorigenic phenotype described in this study (tumorsphere formation), including Herceptin/Lapatinib targeting of HER2, DECMA-1 targeting of sE-Cad, and CADD522 targeting of RUNX2.

Luminal BCs include ductal carcinoma *in situ* (DCIS), which is the most commonly diagnosed BC that accounts for 50% of metastases. Although primary DCIS are easily detected and treated, they are extremely heterogeneous and can recur years after treatment. Transcriptional activation is one mechanism generating heterogeneity, maintaining a tumor-initiating (stem cell) phenotype, and promoting metastasis [62]. The RUNX2 transcription factor is expressed in luminal BCs as well as cell lines and appears to support a stem-like and oncogenic phenotype [20]. Previous studies suggested that RUNX2 can interact with Hippo pathway signaling cofactors to promote anchorage-independent growth [23]. Therefore, to test the hypothesis that RUNX2 promotes an oncogenic phenotype by cooperating with the Hippo tumor suppressor pathway transcriptional modulator TAZ, several luminal BCs were examined. RUNX2 expression supported a TGFβ-driven oncogenic signaling pathway that involves TAZ-mediated activation of tumorsphere formation, the production of soluble E-Cadherin (sE-Cad), and cooperation with HER2, which was increased in RUNX2 expressing cells. The data indicate that sE-Cad cooperates with HER2 and sensitizes BC cells to HER2-targeted drugs. These luminal BC cells expressing RUNX2 appear to rely on HER2 signaling to potentiate their tumorigenic phenotype. The effects on tumorsphere formation are consistent with known roles for RUNX2 and TAZ in tumor-initiating functions. These results have identified several therapeutic targets that converge on RUNX2:TAZ transcriptional regulation in BC cells. The combined signaling pathways may be responsible for a transcriptional program that mediates BC tumorsphere formation and anchorage-independent growth (Figure 6). A better understanding of how these components promote oncogenesis may lead to the development of better therapeutic agents to target multiple components of this signaling cascade to inhibit metastatic disease.

The function of RUNX2 in early stage luminal BC is not completely understood, although it is known to exhibit either tumor suppressive or oncogenic activity, depending on expression of ER [63]. The *RUNX2* gene and protein are upregulated in early stage BC tissue [7, 8, 18, 20, 64] and there is evidence for a luminal BC sub-population that expresses RUNX2 [20]. There are many possible approaches to target transcription factors for cancer therapy [65]. Using a new RUNX2-targeting agent that was designed to inhibit RUNX2 DNA binding [52, 66], we took a direct approach to inhibiting RUNX2 in BC cells. Although attached cells were not affected (data not shown), cells in suspension were sensitive to RUNX2 targeting. The CADD522 compound reduced the levels of RUNX2 protein in two different BC cells and prevented the accumulation of nuclear TAZ, the production of sE-Cad, and tumorsphere formation. Previous studies had shown that TAZ-regulated tumorigenic events, which silence the Hippo pathway, are activated by TGFβ signaling and TEAD transcription factor interactions [26]. Our results show that TGFβ treatment and RUNX2 induction results in Hippo inactivation, which is characterized by increased TAZ nuclear translocalization and interaction with RUNX2. Since growth in suspension increased with RUNX2 expression, while TAZ knockdown reduced anchorage-independent growth, the results are consistent with a cooperative interaction between RUNX2 and TAZ to promote tumorsphere formation. TAZ is the transcriptional cofactor that interacts with RUNX2 in the bone to mediate osteoblast differentiation [22]. Therefore, it is possible that tumor cells hijack this developmental partnership to prime BC cells for homing, implantation, and survival in the bone. This could account for the high bone metastatic recurrence rate observed clinically in luminal BC cases [67]. TAZ is an active oncogene when it translocates to the nucleus where it can upregulate genes responsible for stem cell maintenance and metastasis. It may also be a viable therapeutic target [68]. Although TAZ knockdown inhibited tumorsphere formation almost completely in RUNX2 positive cells, TAZ also was essential for growth in suspension of MCF7 cells in which RUNX2 was not induced (Figure 3D). This may reflect the endogenous subpopulation of cells that already express RUNX2 [20] and which may also use TAZ as a cofactor to promote tumorsphere formation. In addition, it is possible that in some cells TAZ interacts with other nuclear transcription factors such as TEAD or p73 [27]. However, reduced TAZ nuclear levels in response to CADD drug (Figure 3E) in RUNX2 expressing cells suggest that TAZ may associate poorly with other nuclear factors such as TEAD to mediate nuclear localization when RUNX2 is expressed. Overall these results indicate that Hippo signaling and the RUNX2 cofactor TAZ are involved in anchorage-independent growth and tumorsphere formation in luminal BC cells.

Cell migration and invasion of surrounding stroma contributes to tumor metastasis, which is often accompanied by an EMT. RUNX2 increases the expression of the EMT inducer SNAI2 [57]. Concomitant reduction of epithelial markers such as E-Cadherin and ER define an EMT in some cells [32, 33, 57]. However, loss of E-Cadherin and an EMT is not necessary to observe metastasis [34]. RUNX2 disrupted E-Cadherin cell:cell junctions but did not promote a classic EMT in ER-positive MCF7 cells (Supplementary Figure S1D). E-Cadherin is a known tumor suppressor that promotes cell:cell contacts and is sensitive to proteolysis [34]. While E-Cadherin is a defined tumor suppressor that is inactivated by transcriptional or promoter methylation mechanisms [69], the shed soluble ectodomain (sE-Cad) exhibits oncogenic activity [61] and its levels increased in RUNX2+ cells. EGTA (a calcium chelator used to disrupt E-Cadherin hemophilic adhesion) or treatment with the broad spectrum MMP inhibitor o-Phenanthroline inhibited sE-Cad formation indicating that cell:cell adhesion and proteolytic processing is necessary for production and retention of sE-Cad on the cell surface. RUNX2 overexpressing cells were characterized by increased MMP and matrix gene expression (Supplementary Figure S1E), some of which are known E-Cadherin generators (MMP2) and others which have not been shown to mediate proteolytic processing of E-Cadherin (MMP11, MMP12, and MMP16). Therefore, in RUNX2 overexpressing cells an MMP-driven mechanism might be responsible for sE-Cad production. Whether these MMPs directly regulate sE-Cad in luminal BC cells will require further mechanistic studies. DECMA-1 neutralizing antibody targets both full-length E-Cadherin and the sE-Cad ectodomain. In RUNX2 Tet.OFF cells, DECMA-1 increased tumorsphere size in low RUNX2 expressing cells, consistent with other findings that neutralizing antibodies targeting E-Cadherin stimulate MCF7 growth [58] by inhibiting its tumor suppressor function. Blocking full-length E-Cadherin in RUNX2 control cells could have also resulted in a functional EMT leading to the observed increase in tumorsphere size [70]. However, DECMA-1 also inhibited RUNX2 overexpressing MCF7 cells resulting in smaller tumorspheres, consistent with inhibition of the oncogenic sE-Cad ectodomain. Since CADD522 treatment increased sE-Cad levels after 24 hr treatment (Figure 4D), the results are consistent with reduced sE-Cad turnover or shedding in the presence of the RUNX2 inhibitor, perhaps as an adaptive response. However, the eventual decline in sE-Cad levels with CADD522 treatment could be the result of lower RUNX2 protein and transcriptional activity, which may be essential for promoting MMP driven sE-Cad generation.

HER2 is amplified in a subset of ER−/PR− BCs [1, 2, 60] and has also been shown to be expressed in a subset of luminal BC cells in the absence of gene amplification [6]. The ErbB/HER2 receptor family is one of the main targets of sE-Cad signaling and HER2 interacts with sE-Cad to promote ligand-independent cell signaling [59]. HER2-targeting, therefore, may also inhibit tumors that shed the E-Cadherin ectodomain, sE-Cad, by inhibiting downstream signaling initiated through this ligand-independent HER2 receptor activation. HER2 was expressed (non-amplified) in MCF7 cells and MCF7 cells expressing RUNX2 were sensitive to HER2-targeting agents. These data indicate that HER2/ErbB2 is, therefore, functional in RUNX2 overexpressing MCF7 cells. Ithimakin and colleagues used a series of luminal cell lines, mouse xenograft experiments, and patient tumor data to show that HER2-selective agents (trastuzumab) may have clinical benefit among a broad group of patients, even those without *HER2* gene amplification [6]. Furthermore, it was found that BC cells expressed HER2 in metastatic bone lesions without the presence of amplification similar to what is observed in patients with RUNX2 and TAZ expressing tumors [9–11, 29, 30]. In summary, current results support a model consistent with production of sE-Cad in RUNX2 expressing cells and cooperation with HER2 to promote a tumorigenic phenotype. Therefore, RUNX2 and sE-Cad could be potential biomarkers for a currently undetectable subpopulation of cells within luminal BCs that express TAZ and HER2. This subpopulation could benefit from RUNX2 and HER2-targeting agents, which may be a more effective treatment modality in preventing recurrence and metastasis to the bone years after initial diagnosis and treatment.

## MATERIALS AND METHODS

### Cell culture

The MCF7 BC cell line with inducible RUNX2 expression (ER+ MCF7) was prepared using the BD™ Tet-Off System (BD Biosciences). MCF7-RUNX2 cells are ER+ and express wild type p53, PTEN, c-myc, and ras, but do not express p16. MCF7 cells containing tTA (Tetracycline-controlled transactivator) regulatory vector (G418 resistant) were purchased from Clontech (Mountain View, CA), infected with retroviral vectors expressing Flag-tagged RUNX2, and selected with 200 μg/ml hygromycin B. Cells were frozen within three passages and maintained in DMEM (Corning) containing 10% Tet-Approved FBS (Clontech) and the antibiotics G418 (100 μg/ml; Sigma), hygromycin B (200 μg/ml; Roche), and doxycycline (2 μg/ml; Sigma) to repress RUNX2 expression (+Dox). To express RUNX2, cells were grown in the same media but in the absence of doxycycline (−Dox) for 72 hours to achieve maximal RUNX2 protein levels. T47D and HCC1428 luminal BC cells were obtained from ATCC (Manassas, VA) and were a kind gift from Dr. Stuart Martin (University of Maryland). They were maintained in RPMI (Corning) containing 10% FBS (Gemini; #100-106) with 1% Pen/Strep (Gemini; #400-109). T47D luminal BC cells were also supplemented with 0.2 Units/mL bovine insulin (Sigma; #I0516). To validate EMT markers, the triple negative Hs578t BC cells were obtained from ATCC (Manassas, VA) and maintained in DMEM (Corning) supplemented with 5% FBS (Gemini) and 1% Pen/Strep (Gemini). NIH3T3 cells were passaged less than 20 times and maintained in DMEM (Corning) supplemented with 10% FBS (Gemini) and 1% Pen/Strep (Gemini).

### Suspension culture/tumorsphere formation

To measure anchorage-independent growth, MCF7-RUNX2 Tet.OFF cells were grown in the presence (+Dox, RUNX2 negative) or absence (−Dox, RUNX2 positive) of doxycycline for 3 days. Cells were then scraped and counted. 60,000 cells were plated in each well of a 6-well ultra-low attachment plate (Corning; #3471) in Promocell Basal Medium (Promocell; c-22211) complete with Supplement Mix (Promocell; c-39216). Cells were treated with or without 2 ng/mL TGFβ (R&D Systems; #240-B-002). After growth for 10–15 days, cells were photographed and tumorsphere diameters were measured from photographic images (mm). Colony diameters were calculated using the formula: (L+W)/2. Representative photographs were obtained at 4X magnification. Other treatments included: 50 μM CADD522 (ChemBridge Corporation; 5221975), 20 μg/mL DECMA-1 (Sigma-Aldrich; U3254), 10 μg/mL Herceptin (University of Maryland Marlene and Stuart Greenebaum Cancer Center) replenished every 2–3 days, and 1 μM Lapatinib (gift from Dr. Anne Hamburger at the University of Maryland, Baltimore). For TAZ siRNA knockdown experiments, TAZ siRNA was transfected (as below) into MCF7-RUNX2 Tet.OFF cells and 24 hr later cells were scraped and placed into suspension as described above.

### Western blot and antibody protocols

MCF7-RUNX2 Tet.OFF cells were grown to subconfluence in the presence (RUNX2 negative) or absence (RUNX2 positive) of doxycycline for 72 hr in full media as described above. Cells were then treated in minimal DMEM (Sigma, D5030) containing 0.1%BSA, 1% L-glutamine, 2% Tet-Approved FBS, and 1 mM glucose for 16 hours followed by treatment with 2 ng/mL TGFβ (R&D Systems, #240-B-002) for 48 hours in the presence or absence of EGTA to examine sE-Cad expression levels, TAZ localization, and HER2 expression levels. Cells were washed with PBS and scraped from plates. Cytoplasmic and nuclear lysates were obtained using the Low/High Salt extraction method [66]. Cytoplasmic extracts were obtained by resuspending cells in NP40 containing Hypotonic Buffer (10 mM HEPES pH 7.4, 1.5 mM MgCl_2_, 10 mM KCl, 0.5% NP40) followed by 30 min incubation on ice and centrifugation. Nuclear extracts were obtained by resuspending the nuclear pellet in an equal volume of low salt buffer (10 mM HEPES, 25% glycerol, 1.5 mM MgCl_2_, 20 mM KCl, 0.2 mM EDTA) followed by high salt buffer (10 mM HEPES, 25% glycerol, 1.5 mM MgCl_2_, 800 mM KCl, 0.2 mM EDTA) followed by vortex, 30 min incubation on ice, another vortex, and centrifugation. Samples were resolved on 4–12% Bis-Tris polyacrylamide gradient gels (Invitrogen) and transferred to PVDF membranes (Millipore, IPVH00010). Membranes were probed with antibodies listed below followed by development with enhanced ECL (Millipore, WBKLS0500). Proteins were visualized using antibodies recognizing: E-Cadherin (Abcam, HECD-1, ab1416), YAP/TAZ (Cell Signaling, D24E4, #8418), FLAG antibody (Dr. Chen-Yong Lin at Georgetown University, Washington DC), RUNX2 (Cell Signaling, D1L7F, #12556), HER2 (Santa Cruz, C-18, sc-284), ER-α (Santa Cruz, G-20, sc-544), N-Cadherin (Abcam, ab18203), Vimentin (Santa Cruz, V9, sc-6260), Histone H2A (Cell Signaling, #2578), β-actin (Sigma/Aldrich), GAPDH (Cell Signaling, 14C10, #2118), and YAP1 (Novus Biologicals, NB110-58358). Protein levels were normalized to actin and quantified using NIH Image-J software.

### Luciferase assays

NIH3T3 cells were cultured in Costar 24-well plates (Corning Incorporated, Corning, NY; #3524) with 30,000 cells per well and allowed to recover for 24 hours. Cells were then transfected with 100 ng of either RUNX2 expressing plasmid or Tag2a empty vector using X-tremeGENE HP DNA transfection reagent (Roche Life Sciences, Indianapolis, IN; #06 366 236 001). The RUNX2 promoter-luciferase reporter plasmid, 6xOSE2.Luciferase (100 ng) and 10 ng of pTK Renilla were also transfected into the NIH3T3 cells with X-tremeGENE. Cells were treated with 5 μM, 10 μM, or 25 μM CADD522 at the time of transfection for 24 hours. Luciferase assays were performed using the Promega Dual-Glo^®^ system (Madison, WI; #E1960), and luciferase activity was measured in 96-well plates with the Berthold Centro LB 960 Microplate Luminometer (Berthold Technologies, GE). Data were analyzed and normalized to Renilla luciferase and pGL3.Luc control vector. Background luciferase values from Tag2a empty vector transfection were subtracted from RUNX2 expressing NIH3T3 cells. The NFκB.Luciferase promoter-reporter plasmid was a kind gift from Dr. Hancai Dan (Department of Pathology) at the University of Maryland, Baltimore. NIH3T3 cells were transfected with NFκB.Luciferase (100 ng) and 10 ng pTK Renilla in 24-well plates at 30,000 cells/well using X-tremeGENE transfection reagent. Cells were treated overnight with 20 ng/mL TNFα (Cell Signaling, #8902SC) to activate NFκB signaling with or without 10 μM CADD522 and luciferase activity was measured. Data were analyzed and normalized to Renilla and pGL3.Luc control vector.

### Immunoprecipitation/co-immunoprecipitation assay (IP/Co-IP)

Conditioned media were collected from MCF7 cells cultured in the presence (RUNX2 negative) or absence (RUNX2 positive) of doxycycline in minimal DMEM (Sigma, D5030) containing 0.1% BSA, 1% L-glutamine, 2% Tet-Approved FBS, and 1 mM glucose for 16 hours followed by treatment with 2 ng/mL TGFβ (R&D Systems, 240-B-002) for 48 hours. Conditioned media were carefully removed from cells and remaining cellular debris was removed by centrifugation. Conditioned media protein levels were estimated using the Bradford assay. 200 μg of protein was suspended in 200 μL Co-IP buffer (50 mM Tris pH 7.5, 150 mM NaCl, 1 mM EDTA, 1 mM EGTA, 1% Triton X-100, 0.5% NP-40) and pre-cleared in 20 μL of a 50% slurry of Protein G-Sepharose (GE Healthcare, 17-0618-01) for 30 minutes. Pre-cleared supernatants were then incubated overnight with 0.5 μg of E-Cadherin antibody (Abcam, HECD-1, ab1416). Protein G-Sepharose was added for 1 hour and the precipitated complexes were washed with Co-IP buffer. Proteins were eluted from the beads using 0.1 M Glycine buffer (pH 2.4), treated with 1X SDS loading buffer containing β-mercaptoethanol, and heated at 97°C for 10 min. Samples were resolved on a 4–12% Bis-Tris polyacrylamide gel (Invitrogen) and transferred to PVDF membranes (Millipore). Immunoblots were probed for E-Cadherin (Abcam, HECD-1, ab1416) followed by development with enhanced ECL (Millipore).

To test for RUNX2 and TAZ protein interaction, nuclear lysates were obtained using NucBuster (Novagen, #71183-3) from MCF7 cells grown in the presence (RUNX2 negative) or absence (RUNX2 positive) of doxycycline in minimal DMEM (Sigma, D5030) containing 0.1%BSA, 1% L-glutamine, 2% Tet-Approved FBS, and 1 mM glucose and 2 ng/mL TGFβ for 4 and 24 hours. Briefly, 400 μg of protein was resuspended in Co-IP buffer to a final volume of 200 μL. Lysates were precleared with 35 μL of a 50% slurry of Protein G-Sepharose (GE Healthcare) for 1 hr. Pre-cleared nuclear lysates were then incubated with 4 μL of YAP/TAZ antibody (Cell Signaling) overnight. Protein G-Sepharose was added for 1 hour and the precipitated complexes were washed with Co-IP buffer. Proteins were eluted from the beads using 0.1 M Glycine buffer (pH 2.4), treated with 1X SDS loading buffer containing β-mercaptoethanol, and heated at 97°C for 10 min. Samples were resolved on a 4–12% Bis-Tris polyacrylamide gel (Invitrogen) and transferred to PVDF membranes (Millipore). Immunoblots were probed for RUNX2 (Cell Signaling, D1L7F, #12556), and YAP/TAZ (Cell Signaling, D24E4, #8418) followed by development with enhanced ECL (Millipore). To visualize the TAZ protein band a conformation specific rabbit secondary antibody was used (Cell Signaling, L27A9, #5127). Rabbit IgG and beads alone were used as Co-IP controls.

### siRNA mediated knockdown of TAZ

TAZ knockdown was performed in MCF7-RUNX2 Tet.OFF cells using Custom 23 mer desalted siRNA oligonucleotides from Sigma and a Universal Scrambled Negative Control siRNA Duplex from Origene (Catalog No. SR30004): TAZ siRNA #1: GACAUGAGAUCCAUCACUAUU; TAZ siRNA #2: GGACAAACACCCAUGAACAUU; TAZ siRNA #3: AAGCCUAGCUCGUGGCGGAUU. Briefly, MCF7-RUNX2 Tet.OFF cells were grown in the presence (RUNX2 negative) or absence (RUNX2 positive) of doxycycline for 3 days and then transfected with corresponding siRNA using Lipofectamine-2000 (Invitrogen, #1168-019). RIPA extracts were obtained 48 hr post transfection and total protein was analyzed by Western blot (see above) and probed for TAZ protein expression. Protein levels were normalized to actin and quantified using NIH Image-J software.

To assay for sE-Cad levels, MCF7-RUNX2 Tet.OFF cells were grown in the presence (RUNX2 negative) or absence (RUNX2 positive) of doxycycline for 3 days and then transfected with TAZ siRNA #1 using Lipofectamine-2000. Cells were trypsinized, replated 24 hours later, and allowed to reattach. After 72 hours, nuclear and cytoplasmic extracts were obtained using the High/Low salt extraction method described above and analyzed for sE-Cad, TAZ, and RUNX2. Protein levels were normalized to actin and quantified using NIH Image-J software.

### CADD522 drug treatments

The CADD522 compound (MF = C_15_H_13_Cl_2_NO_3_) has a molecular weight of 326.175, a high LogP value of 3.25 (logarithm of its partition coefficient between n-octanol and water, a measure of the compound's hydrophilicity with low hydrophilicity = high LogP), a low LogSW of −4.35 (measure of aqueous solubility), three rotatable bonds, a hydrogen bonding donor/acceptor ratio of 2/3 (Hdon/Hacc), a polar surface area of 66.4 (tPSA; indicative of good cell membrane permeability), and an IC50 of 10 nM in D-ELISA DNA binding assays [52]. MCF7-RUNX2 Tet.OFF cells were pretreated in the presence (RUNX2 negative) or absence (RUNX2 positive) of doxycycline for 3 days. RUNX2 transiently transfected T47D or HCC1428 breast cancer cells were grown in media that was then replaced with full media (as listed in cell culture section) and treated with or without 50 μM CADD522. Cells were allowed to grow for 24, 48, and 72 hours. Nuclear and cytoplasmic extracts were obtained using the High/Low salt extraction method and protein levels were analyzed by Western blot (see above).

### Attachment and invasion assay

Tissue culture plates were coated with Fibronectin (1 μg/ml), extracellular matrix (ECM; from endothelial cells cultured to confluence and treated with 5 mM EDTA to remove cells), or with confluent endothelial cells (human bone marrow endothelial cells). MCF7-RUNX2 Tet.OFF cells were grown in the presence (+Dox, RUNX2 negative) or absence (−Dox, RUNX2 positive) of doxycycline for 3 days and were then added to the indicated plates for 120 min in minimal media (Sigma, D5030) containing 0.1% FBS, 5 mM glucose, and 2 ng/ml TGFβ. The number of cells/field attached to Fibronectin, ECM, or endothelial monolayer was counted from 3–4 fields/well.

### TCGA RUNX2 protein analysis

The Cancer Genome Atlas (TCGA) data were obtained from the online cbioportal (http://www.cbioportal.org/public-portal). The results shown represent protein expression and are based upon data generated by the TCGA Research Network (http://cancergenome.nih.gov). Briefly, cellular proteins were extracted and denatured in SDS sample buffer. After serial dilution of each sample, cell lysates were arrayed on nitrocellulose-coated slides and probed with specific RUNX2 and HRP-coupled antibodies to detect a signal by DAB colorimetric reaction. Spot densities were determined by MiroVigene (automatic spot finding and background subtraction) and protein concentrations were determined by super curve fitting and normalized for protein loading.

### SuperArray gene expression analysis

MCF7 Tet.OFF cells were grown for 3 days in the presence (RUNX2 negative) or absence (RUNX2 positive) of doxycycline in complete media. Cells were washed once with 1X PBS and glucose starvation media was added (D5030 + 2% FBS + 1 mM glucose) and maintained for 16 hrs. TRIzol (Ambion; #15596018) extraction was performed to obtain total RNA. RNA was suspended in nuclease-free Molecular grade water (HyClone Laboratories, Logan, UT; SH30538.01). cDNA was synthesized using the RT2 First Strand Kit (Qiagen; #330401) followed by real-time QPCR on the Applied Biosystem model 7300 thermocycler using the RT2 Profiler PCR Array for Human Extracellular Matrix and Adhesion Molecules (Qiagen; 330231 PAHS-013ZA). Data were analyzed using RT2 Profiler PCR Array Data Analysis v3.5 available online through Qiagen.

### Statistics

Statistical analyses were performed using Student's *t*-test and ANOVA analysis to determine statistical significance between RUNX2 positive treatment groups or RUNX2 negative treatment groups. Results were considered significant when *p* < 0.05.

## SUPPLEMENTARY FIGURE


