# Supplementary material for: RUNX2 and TAZ-dependent signaling pathways regulate soluble E-Cadherin levels and tumorsphere formation in breast cancer cells
Source: Oncotarget. 2015 Jul 20;6(29):28132–50. doi: 10.18632/oncotarget.4654 (PMC4695049; doi:10.18632/oncotarget.4654)
Supplement: Supplementary file 1 [file oncotarget-06-28132-s001.pdf]

SUPPLEMENTARY FIGURE

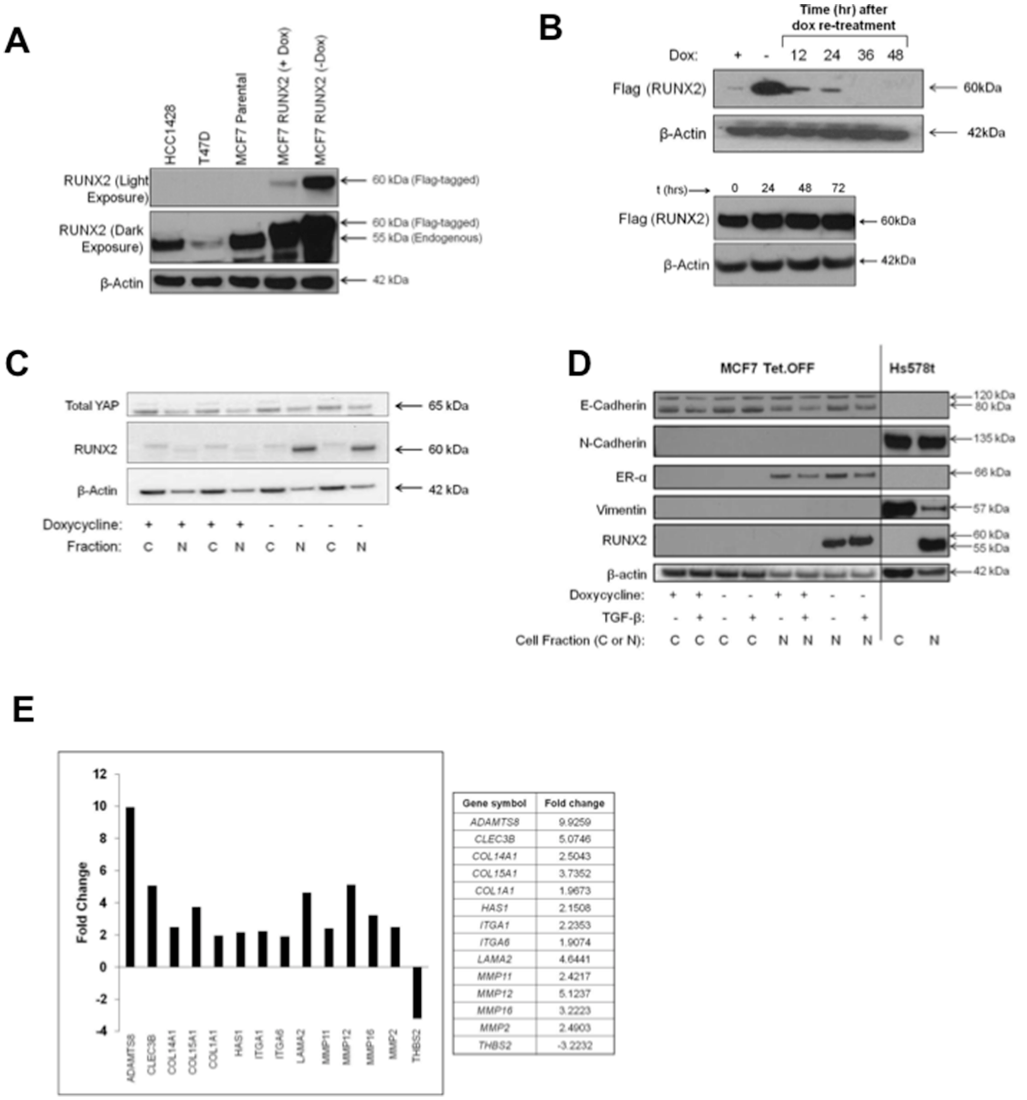

Gene symbol	Fold change
ADAMTS8	9.9259
CLEC3B	5.0746
COL14A1	2.5043
COL15A1	3.7352
COL1A1	1.9673
HAS1	2.1508
ITGA1	2.2353
ITGA6	1.9074
LAMA2	4.6441
MMP11	2.4217
MMP12	5.1237
MMP16	3.2223
MMP2	2.4903
THBS2	-3.2232

Supplementary Figure S1: RUNX2 expression in luminal BC. A. Endogenous RUNX2 expression in MCF7 Parental, T47D, and HCC1428 BC cells. Nuclear protein fractions were obtained using the High/Low salt extraction method. Cells were grown in full media (DMEM for MCF7 parental; RPMI for T47D and HCC1428) and then fractionated. Proteins were resolved by SDS-PAGE and RUNX2 protein bands were visualized using a RUNX2 specific antibody (Cell Signaling) to detect endogenous RUNX2 and FLAG-tagged RUNX2. Light and dark exposures were obtained to visualize endogenous RUNX2 protein levels next to over-expressing MCF7-RUNX2 Tet.OFF cells (RUNX2+). **B.** Repression of RUNX2 expression in MCF7-RUNX2 Tet.OFF cells after retreatment with doxycycline. MCF7-RUNX2 Tet.OFF cells were grown in the presence (RUNX2–) or absence (RUNX2+) of doxycycline for 72 hr and RUNX2 expression was analyzed by Western blot. For some cells, retreatment with doxycycline was performed for 12, 24, 36 or 48 hr to repress RUNX2 expression (*top panel*). RUNX2 levels were also analyzed over 72 hr in RUNX2 overexpressing MCF7 cells not treated with CADD522 (*bottom panel*). **C.** YAP expression and localization are not affected by RUNX2 in MCF7 cells. MCF7-RUNX2 Tet.OFF cells were cultured in the presence (RUNX2–) or absence (RUNX2+) of doxycycline and analyzed for changes in YAP localization using specific antibodies. YAP was detected in both cytoplasmic and nuclear fractions of both RUNX2 overexpressing and control cells. **D.** RUNX2 does not promote an EMT. MCF7-RUNX2 Tet.OFF cells were grown in the presence (RUNX2–) or absence (RUNX2+) of doxycycline and nutrient deprived for 16 hours in D5030 media containing 2% FBS and 1 mM glucose. Cells were then treated for 48 hours with 2 ng/mL TGFβ. Nuclear and cytoplasmic fractions were obtained and proteins analyzed by western blot. E-Cadherin, N-Cadherin, ER-α, vimentin, RUNX2, and β-actin protein levels were analyzed. Hs578t nuclear and cytoplasmic extracts were analyzed by Western blot and used as antibody controls for mesenchymal markers. **E.** RUNX2 increases the expression of numerous MMP and extracellular matrix genes. MCF7 Tet.OFF cells were grown for 3 days in the presence (RUNX2–) or absence (RUNX2+) of doxycycline in complete media. Cells were washed once with PBS and glucose starvation media was added (D5030 + 2% FBS + 1 mM glucose) and maintained for 16 hrs. RNA was isolated, cDNA was synthesized and real-time QPCR was performed using the RT2 Profiler PCR Array for Human Extracellular Matrix and Adhesion Molecules. Fold-change is shown relative to control MCF7 cells (+doxycycline) expressing low RUNX2.
